# Pro-inflammatory miR-223 mediates the cross-talk between the IL23 pathway and the intestinal barrier in inflammatory bowel disease

**DOI:** 10.1186/s13059-016-0901-8

**Published:** 2016-03-30

**Authors:** Huiling Wang, Kang Chao, Siew Chien Ng, Alfa Hc Bai, Qiao Yu, Jun Yu, Manying Li, Yi Cui, Minhu Chen, Ji-Fan Hu, Shenghong Zhang

**Affiliations:** Division of Gastroenterology, The First Affiliated Hospital, Sun Yat-sen University, No. 58, Zhongshan Road 2, Guangzhou, 510080 P.R. China; Department of Medicine and Therapeutics, State Key Laboratory of Digestive Disease, Institute of Digestive Disease, Li Ka Shing Institute of Health Science, Hong Kong, P.R. China; Stem Cell and Cancer Center, First Hospital, Jilin University, Changchun, P.R. China; Stanford University Medical School, Palo Alto Veterans Institute for Research, Palo Alto, CA 94304 USA

**Keywords:** Crohn’s disease, Ulcerative colitis, Interleukin 23, miRNA, Pathway

## Abstract

**Background:**

The IL23/Th17 pathway is essential for the onset of inflammatory bowel disease (IBD), yet the specific mechanism by which this pathway initiates the disease remains unknown. In this study, we identify the mechanisms that mediate cross-talk between the IL23 pathway and the intestinal barrier in IBD.

**Results:**

The downstream targets of the IL23 pathway were identified by RNA array profiling and confirmed by immunohistochemical staining. The role of miRNAs that interact with IL23 was explored in mice with TNBS-induced colitis. Claudin-8 (CLDN8), a multigene family protein that constitutes the backbone of tight junctions, was identified as a novel target of IL23 in IBD. CLDN8 was significantly downregulated in IBD patients with inflamed colonic mucosa, and in trinitrobenzene sulphonic acid (TNBS) induced colitis in mice. Therapeutic treatment of colitis in mice using an IL23 antibody restored CLDN8 abundance, in parallel with recovery from colitis. In addition, we identify miR-223 as a novel mediator of the crosstalk between the IL23 signal pathway and CLDN8 in the development of IBD. MiR-223 was upregulated in IBD, and its activity was regulated through the IL23 pathway. Antagomir inhibition of miR-223 reactivated CLDN8 and improved a number of signs associated with TNBS-induced colitis in mice.

**Conclusions:**

Our study characterizes a new mechanistic pathway in IBD, in which miR-223 interacts with the IL23 pathway by targeting CLDN8. Strategies designed to disrupt this interaction may provide novel therapeutic agents for the management of IBD.

**Electronic supplementary material:**

The online version of this article (doi:10.1186/s13059-016-0901-8) contains supplementary material, which is available to authorized users.

## Background

Inflammatory bowel disease (IBD) comprises two distinct phenotypes: ulcerative colitis (UC) and Crohn’s disease (CD), each of which has unique clinical manifestations while sharing many genetic and mechanistic features [1, 2]. In the past decade, the incidence of IBD in Asia has increased dramatically. Our recent population-based study showed that Guangzhou and Hong Kong are among the top three cities in Asia with the highest incidence of IBD at 3.44 and 3.06 per 100,000, respectively [3]. Although the exact pathophysiology of IBD is not fully understood, the etiology of this disease is known to be multifactorially driven by a number of genetic and environmental factors, including loss of regulation of the host’s innate immune response and defects in mucosal barrier function [4].

The intestinal epithelial barrier is crucial for maintaining the intestinal homoeostasis because of its location between the luminal bacteria and the host’s innate immune system. This epithelial barrier represents the first exposure to various external environmental factors, which can trigger the onset of various diseases, including IBD [5]. Tight junctions (TJs) are the main components of the intestinal epithelial barrier, and they function primarily in controlling cellular polarity and adhesion [6]. The components of the tight junction include Occludin, Tricellulin [7], the junctional adhesion molecule (JAM) proteins [8], and the large Claudin family. The Claudins, consisting of 24–27 members in mammalian genomes, are the major determinant of electrolyte permeability through the paracellular pathway, and are regarded as the backbone of the intestinal barrier [9]. Claudins have been reported to be dysregulated in IBD patients.

Recent studies have found that the IL23/Th17 axis is involved in the regulation of IBD [10, 11]. Inhibition of the pathway by anti-IL23P19 monoclonal antibody attenuated Trinitrobenzene sulfonic acid (TNBS)-induced Crohn’s disease in rats [12]. Although both the dysfunction of intestinal barrier properties and the IL23/Th17 pathway are key contributors to the onset of IBD, it is still not clear whether there exists a link between these two factors in mediating the chronic inflammation of IBD.

MiRNAs are small non-coding RNAs that regulate gene expression by base pairing with target mRNAs at the 3’-untranslated region, leading to mRNA cleavage and translational repression [13, 14]. It has been suggested that miRNAs regulate tens or hundreds of targets [15], and a number of biological processes are regulated by miRNAs, including cell proliferation, cell death, stress resistance, and differentiation of intestinal epithelial cells [16, 17]. It has also been reported that the expression of miRNAs is abnormal in IBD patients, suggesting that the altered expression of miRNAs may be involved in pathogenesis of IBD [18, 19]. However, the role of miRNAs in the IL23 pathway has not been explored.

Previous studies found that cytokines, including TNF-α, might induce or inhibit the expression of miRNAs [20, 21]. Therefore, we hypothesized that the IL23 pathway might interact with miRNAs to cause dysfunction of the intestinal epithelial barrier. In this study, we sought to identify the downstream targets of the IL23 pathway in the development of IBD, including those miRNAs that mediate the cross-talk between the IL23/Th17 axis and the intestinal epithelial barrier.

## Results

### The role of the IL23 pathway in TNBS-induced colitis

**Fig. 1:**
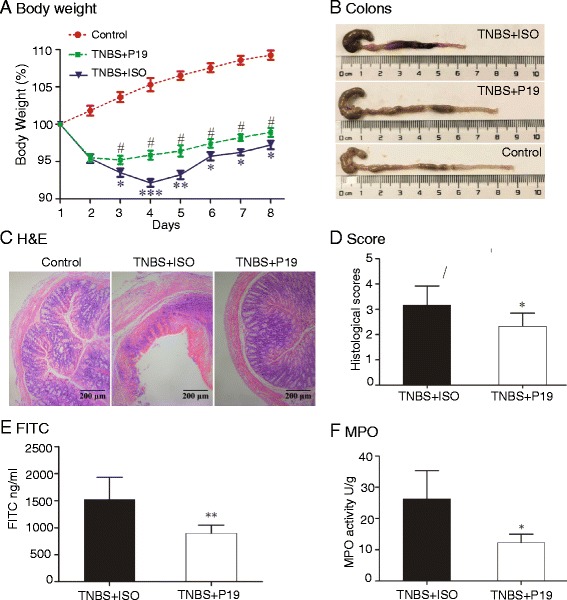
The pathological role of the IL23/Th17 pathway in TNBS-induced colitis. **a** Recovery of body weight in mice with TNBS-induced colitis by anti-IL23P19. Control: colitis mice treated with ethanol; TNBS + P19: TNBS-induced colitis mice treated with an IL23 antibody; TNBS + ISO: TNBS-induced colitis mice treated with an isotype control; *P <0.05, **P <0.01, ***P <0.001 for comparison between the TNBS + ISO and the Ethanol Control; ^#^ *P* <0.05 for the statistical significance between the TNBS + P19 treatment group and the TNBS + ISO control group. **b** Representative images of the colon in treated mice with colitis. **c** Representative cross-sections of the transverse colon. Magnification of the images is 200-fold. **d** Anti-IL23P19 therapy reduces the histological score. *P <0.05 as compared with the TNBS + ISO control group. **e** Serum FITC-dextran was quantified as a measure of intestinal permeability. **P <0.01 as compared with the TNBS + ISO control group. **f** Effects on MPO activity measurement by Anti-IL23P19. *P <0.05 as compared with the TNBS + ISO control group

The IL23/Th17 pathway is critical to the onset of IBD. To delineate the mechanisms underlying the role of IL23 in IBD, we established a colitis model in BALB/c mice using TNBS, and treated these mice with anti-IL23P19 mAb. The animals with colitis that were treated with anti-IL23P19 (TNBS + P19) experienced a significant recovery in body weight compared to the isotype control group (TNBS + ISO) (Fig. 1a). In addition, the anti-IL23P19 treatment improved many cardinal signs of colitis in the animals, including the area of ulceration, a depletion of mucin-producing goblet and epithelial cells, a thickening of the muscular layer, degree of leukocyte and PMN infiltration, as well as histological scores (Fig. 1b-d).

The role of the IL23 pathway in the pathogenesis of IBD was also evaluated by two additional assays. Intestinal permeability was examined using the FITC-labeled dextran assay. We found that the anti-IL23P19 group showed a significantly greater decrease in intestinal permeability to FITC-dextran when compared with the isotype control group (*P* <0.01) (Fig. 1e). Similarly, the colonic myeloperoxidase (MPO) activity, a biochemical assay for acute intestinal inflammation, was significantly alleviated by the anti-IL23P19 treatment (Fig. 1f). Together, these data confirm that targeting this over-reactive pro-inflammatory pathway is an effective therapeutic strategy against IBD as previously reported [22–24].

### Identification of CLDN8 as a novel target gene in IBD

**Fig. 2:**
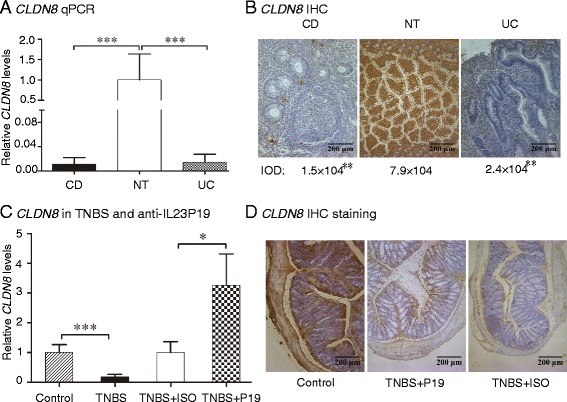
Identification of *CLDN8* as a novel target controlled by the IL23 pathway in IBD patients. **a** Quantitative PCR of *CLDN8* in colonic inflamed mucosa of IBD patients. CD: Crohn’s disease (n = 50); UC: ulcerative colitis (n = 50); NT: normal subjects (n = 50). ***P <0.001 as compared with normal controls. **b** Representative immunostaining of *CLDN8* in IBD-inflamed tissues and normal intestinal. Magnification of the images is 200-fold. IOD: Integrated optical densities of *CLDN8* in colonic inflamed mucosa of IBD patients. **P <0.01 as compared with normal controls. **c** Anti-IL23P19 treatment reverses the downregulation of *CLDN8* in TNBS-induced colitis tissues. *P <0.05, ***P <0.001 as compared with the controls. **d** Representative immunostaining of *CLDN8*

Using microarray analyses in IBD tissues, Fang *et al.* reported that hundreds of genes are altered in IBD tissues, including the CXC chemokine family, SLC16A9, SLC17A4, SLC23A3, and SLC3A1 [25]. To identify molecular targets in the IL23 pathway, we used an RNA microarray chip to screen genes that are differentially expressed between IBD and healthy controls. In this study, we found that there were 353 genes that showed greater than four-fold differential expression (285 upregulated and 68 downregulated) (Additional files 1 and 2: Tables S1 and S2). Among them, claudin-8 (CLDN8), a member of the claudin family proteins that constitute the backbone of the intestinal barrier, was highly expressed in normal tissues, but was downregulated in IBD tissues (Additional file 3: Figure S1A). In clinically collected tissue samples, we confirmed that *CLDN8* was significantly downregulated in patients with CD and UC as compared with that in control patients (Fig. 2a, quantitative PCR; Additional file 3: Figure S1B, western blot). Consistent with these findings, immunohistochemical (IHC) staining also demonstrated that *CLDN8* was significantly reduced in IBD colonic mucosa (Fig. 2b, integrated optical density (IOD), *P* <0.01).

Similarly, in the colitis animal model we observed the downregulation of *CLDN8* in TNBS-induced colitis tissues. Interestingly, treatment with anti-IL23P19 increased *CLDN8* 2.8-fold (Fig. 2c, quantitative PCR, *P* = 0.028; Additional file 3: Figure S1C, western blot). Upregulation of *CLDN8* by anti-IL23P19 was also confirmed in mice with colitis as compared with the isotype controls using IHC staining (Fig. 2d). The Claudin family proteins are required for proper functioning of the intestinal barrier. Dysfunction of the intestinal barrier contributes to the onset of IBD. Our data thus identify *CLDN8* as a novel gene target both in IBD patients and in the anti-IL23P19-treated colitis animal model.

### CLDN8 is required for the maintenance of junction tightness of colonic cells

**Fig. 3:**
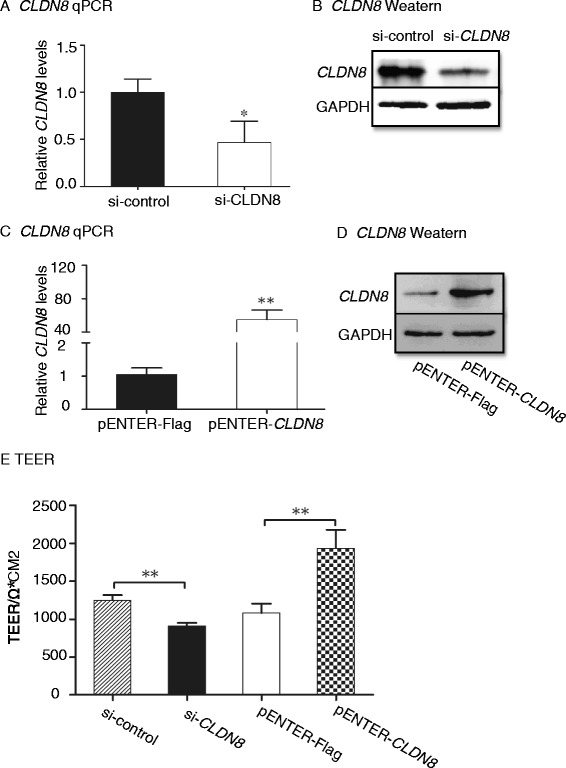
*CLDN8* regulates TEER in Caco-2 cells. **a**, **b**: Knockdown of *CLDN8* by siRNA in Caco-2 cells as quantitated by qPCR (**a**) and western blot (**b**). **c**, **d** Ectopic expression of *CLDN8* in Caco-2 cells as quantitated by qPCR (**c**) and western blot (**d**). **e** *CLDN8* regulates the TEER in Caco-2 cells. *P <0.05, **P <0.01 as compared with the control

Measurement of transepithelial electrical resistance (TEER) is considered to be a good indication of the tightness of junctions between colonic cells. We investigated the role of *CLDN8* by knocking down *CLDN8* using siRNA or overexpressing it by ectopic expression of *CLDN8* in Caco-2 cells (Fig. 3a-d). As compared with the control group (si-control), knockdown of *CLDN8* significantly reduced the TEER. In contrast, ectopic expression of *CLDN8* significantly enhanced the tight junction of epithelial cells (Fig. 3e). Thus, the newly identified *CLDN8* is required for maintaining normal intestinal barrier properties.

### CLDN8 is a novel downstream component of the IL23 pathway

**Fig. 4:**
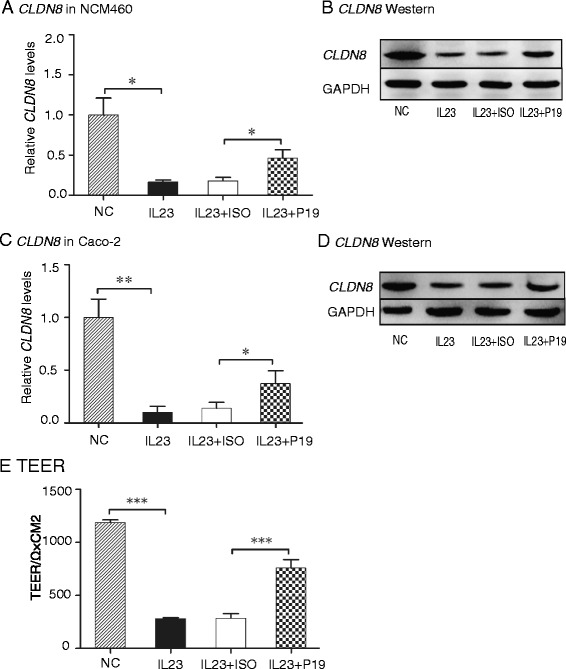
IL23 downregulates *CLDN8* in Caco-2 and NCM460 cells. **a**, **b** IL23 downregulates *CLDN8* in NCM460 cells as quantitated by qPCR (**a**) and western blot (**b**). **c**, **d** IL23 downregulates *CLDN8* in Caco-2 cells as quantitated by qPCR (**c**) and western blot (**d**). **e** IL23 decreases TEER. NC: cells treated with PBS. *P <0.05, **P <0.01, ***P <0.001

We examined the role of IL23 in the regulation of *CLDN8* in two human colonic epithelial cell lines. After treating NCM460 cells with IL23 for 72 h, transcriptional expression levels of *CLDN8* in intestinal epithelial cells were assessed by Q-PCR. *CLDN8* was downregulated by 84 % in the IL23-treated group (*P* = 0.019). However, co-treatment with anti-IL23P19 significantly reduced the reduction of *CLDN8* (*P* <0.05) (Fig. 4a, b). Similar results were also observed in Caco-2 cells (Fig. 4c, d).

We further examined the impact of IL23 on intestinal barrier properties by measuring transepithelial electrical resistance (TEER). Treatment of Caco-2 cells with IL23 resulted in a decrease in TEER by 76.4 % (Fig. 4e). However, co-treatment with anti-IL23P19 significantly attenuated the damage of this intestinal barrier property. Together, these results suggest that the IL23 pathway affects the intestinal barrier property.

### MiR-223 targets CLDN8 in the IL23 pathway

**Fig. 5:**
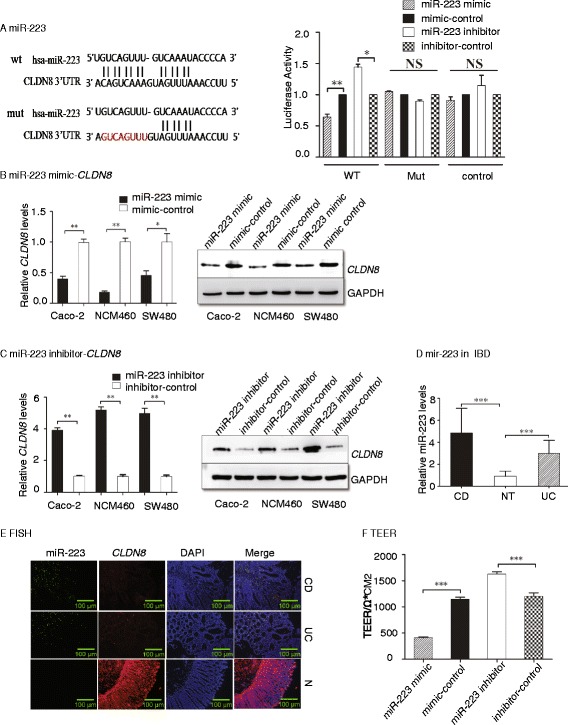
Identification of miR223 in targeting *CLDN8* in colonic epithelial cells. **a** Regulation of the *CLDN8* 3’-UTR reporter by miR-223 mimics or inhibitors. Left panel: Targeting sequences of wild-type (wt) and mutant (mut) 3’-UTR of the human *CLDN8*. The mutated sequences are marked in red. Right panel: The gene activity was quantitated by luciferase assay in 293T cells. WT: wild type 3’-UTR; Mut: mutant 3’-UTR. **b** MiR-223 mimics downregulate *CLDN8* in colonic epithelial cells. Left panel: qPCR; right panel: western blot. **c** MiR-223 inhibitors upregulate *CLDN8* in colonic epithelial cells. Left panel: qPCR; right panel: western blot. **d** Upregulation of miR-223 in UC and CD. **e** Fluorescence *in situ* hybridization of miR-223 and immunostaining of *CLDN8* in human IBD tissues and normal tissues (magnification × 200). **f** MiR-223 regulates TEER. *P <0.05, **P <0.01, ***P <0.001 as compared with the control

We then sought to delineate the mechanism by which IL23 targets *CLDN8* in the dysfunctional intestinal barrier property in IBD. Using microRNA prediction algorithms (www.microRNA.org), we identified miR-223 as a putative candidate microRNA that targets the 3’-UTR of *CLDN8* (Fig. 5a, left panel).

To test the role of miR-223 in regulating the 3’-UTR of *CLDN8*, we constructed a plasmid encoding a firefly luciferase transcript with either the wild-type or a mutant 3’-UTR of *CLDN8*. We found that the miR-223 mimic decreased the expression of the transcript containing the wild-type 3’-UTR of *CLDN8*, while the miR-223 inhibitor increased the expression (Fig. 5a, right panel). However, miR-223 did not cause significant changes in the transcript containing the mutant 3’-UTR of *CLDN8*. These data demonstrate a specific inhibitory effect of miR-223 on the 3’-UTR of *CLDN8*.

We then examined the regulatory effect of miR-223 on endogenous *CLDN8* by transfecting vectors containing miR-223 mimic, inhibitor, or controls into Caco-2, NCM460, and SW480 cells (Additional file 4: Figure S2). MiR-223 mimics inhibited *CLDN8* expression at both the mRNA and protein levels (Fig. 5b, left panel: quantitative PCR; right panel: western blot). On the other hand, when cells were transfected with miR-223 inhibitor, the mRNA transcript and protein level of *CLDN8* were elevated compared with control-transfected cells (Fig. 5c, left panel: quantitative PCR; right panel: western blot). These data indicate that endogenous *CLDN8* is negatively regulated by miR-223 in colonic epithelial cells.

### MiR-223 is negatively correlated with CLDN8 in UC and CD patients

Considering the role of miR-223 in targeting *CLDN8*, we determined whether they were differentially expressed in colonic mucosa of IBD patients. A total of 50 CD and 50 UC colonic mucosa and 50 normal samples were examined. In colonic mucosa of CD patients, the expression of miR-223 was 4.87-fold higher than that in the normal subjects (*P* <0.001, Fig. 5d). Similarly, in the colonic mucosa of UC patients, miR-223 was 2.9-fold higher than that seen in normal colonic mucosa (*P* <0.001).

We then use a FISH assay to compare the expression pattern of mirR-223 and *CLDN8* in colonic mucosa. We found that miR-223 was upregulated, while *CLDN8* was downregulated in colonic mucosa from IBD patients. There was a clear correlation between miR-223 and *CLDN8* in colonic tissues of the IBD patients (Fig. 5e).

The TEER assay was then used to verify the effects of miR-223 mimics and inhibitors on the tightness of junctions between colonic cells. Incubation of the monolayer colonic cells with miR-223 mimics resulted in a decrease in TEER by 64 % compared to the control group. In contrast, miR-223 inhibitors increased the TEER by 35.4 % (Fig. 5f). These results indicate that miR-223 affects the tightness of junctions between colonic cells.

### Therapeutic treatment of TNBS-induced colitis mice

**Fig. 6:**
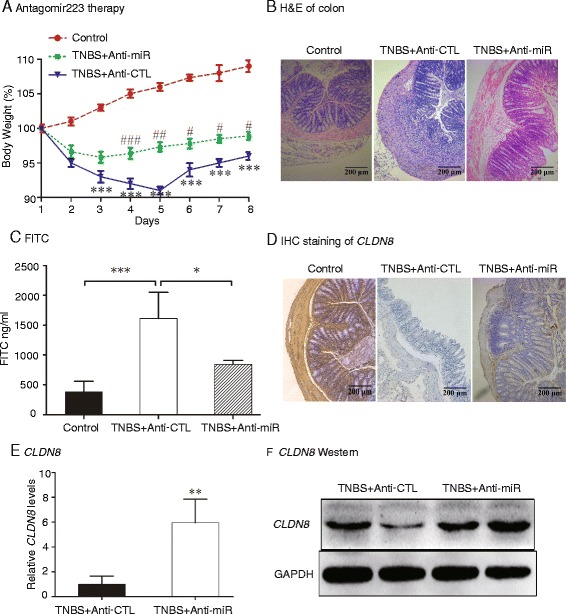
Therapeutic treatment of miR-223 antagomirs in TNBS-induced colitis mice. **a** Recovery of body weight in mice with colitis by miR-223 antagomirs. Control: colitis mice treated with ethanol; TNBS + Anti-miR: colitis mice treated with antagomir223; TNBS + Anti-CTL: colitis mice treated with an isotype control of antagomir223. ***P <0.001 for comparison between the TNBS + Anti-CTL group and the Ethanol Control group; ^#^ *P* <0.05, ^##^ *P* <0.01, ^###^ *P* <0.001 for the statistical significance between the TNBS + Anti-miR group and the TNBS + anti-CTL group. **b** Representative images of the colon (H&E staining). **c** Serum FITC-dextran was quantified as a measure of intestinal permeability. **d**-**f** Antagomir223 upregulates *CLDN8*

To further confirm the role of miR-223 in IBD, we tested the potential of miR-223 antagomir treatment in mice with TNBS-induced colitis. As seen in Fig. 6a, body weights for the antagomir-control (TNBS + Anti-CTL) group were significantly decreased compared to the control group (only treated with ethanol). However, the antagmir223 treatment resulted in significant recovery in body weight compared to the antagomir-control group. Animals with TNBS-induced colitis showed large areas of ulceration, severe depletions of mucin-producing goblet and epithelial cells, thickening of the muscular layer, and high levels of leukocyte and polymorphonuclear (PMN) infiltration. Administration of mirVana® miRNA inhibitors improved the above-mentioned signs and the histological scores (Fig. 6b, Additional file 5: Figure S3B and S3C).

To define the role of mirVana® miRNA inhibitors on mucosal barrier, intestinal permeability was examined in the mice. Mice treated with antagmir223 exhibited a significantly greater decrease in intestinal permeability to FITC-dextran as compared to the antagomir-control group (*P* <0.01) (Fig. 6c). Similarly, the antagomir-223 therapy reduced acute intestinal inflammation as measured by colonic myeloperoxidase (MPO) activity (Additional file 5: Figure S3D).

By inhibiting miR-223, the mirVana® miRNA inhibitor reactivated *CLDN8* in colitis tissues (Fig. 6d-f, *P <*0.01), in parallel with the recovery in loss of body weight, and improvement of histological appearance, histological score and MPO activity, and the preservation of the integrity of the intestinal epithelial barrier.

### Crosstalk between IL23, MiR-223, and CLDN8 in the development of IBD

**Fig. 7:**
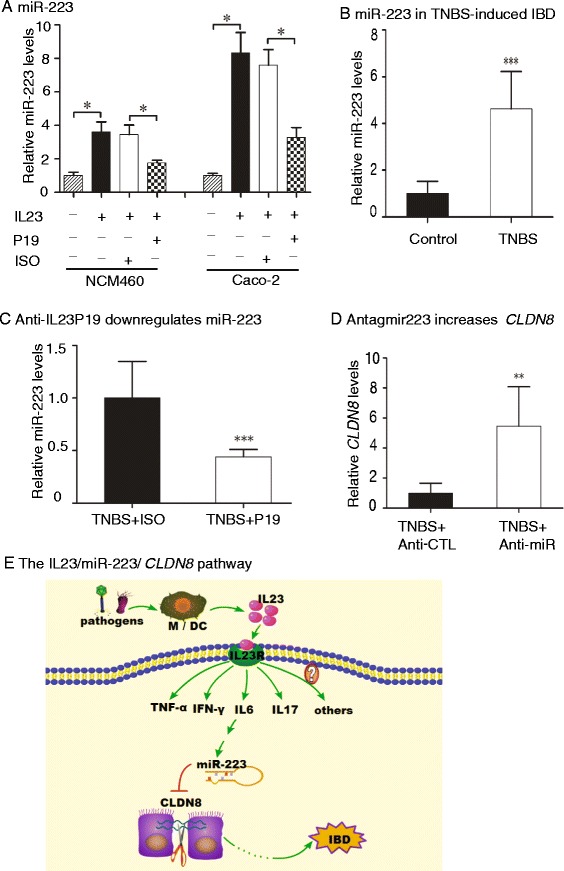
Interaction among IL23, miR223 and *CLDN8*. **a** IL23 controls the expression of miR-223 in NCM460 and Caco-2 cells. NCM460 is a human colon mucosal epithelial cell line. **b** miR-223 is upregulated in the colonic mucosa of mice with TNBS-induced colitis. **c** The anti-IL23P19 therapy reduces the expression of miR-223. **d** Antagomir223 upregulates the RNA expression of *CLDN8*. **e** The proposed IL23/miR-223/*CLDN8* pathway. Activation of the IL23 cascade upregulates the pro-inflammatory miR-223. By targeting *CLDN8*, miR-223 impairs intestinal barrier, leading to the development of IBD. M: Macrophage; DC: Dendritic cells. *P <0.05, **P <0.01, ***P <0.001 as compared with the control

Given the importance of miR-223 in IBD, we further examined if the microRNA was also controlled by the IL23 pathway. Two colonic epithelial cell lines (NCM640 and Caco-2) were treated with IL23 for 72 h. The expression of miR-223 and *CLDN8* were assessed by Q-PCR. Compared to the control, the expression miR-223 was increased by 3.6-fold in NCM460 cells (*P* <0.05, Fig. 7a). However, co-treatment with anti-IL23P19 attenuated the activation of miR-223, compared with the isotype-control (*P* <0.05). Similar data were also observed in Caco-2 cells (Fig. 7a).

We then examined the regulation of miR-223 by IL23 in mice with TNBS-induced colitis. MiR-223 was upregulated in the colonic mucosa of TNBS-induced colitis mice (Fig. 7b). However, treatment of these animals with anti-IL23P19 reduced the miR-233 by 56 % (*P* <0.001, Fig. 7c).

By reducing miR-223 with the antagomir, we observed a parallel increase of *CLDN8* in the colitis model (Fig. 7d). Collectively, these data suggest that miR-223 interacts with the IL23/Th17 pathway and the target gene*-CLDN8* in IBD (Fig. 7e).

## Discussion

IL23 is a crucial factor in the manifestation of intestinal inflammation in IBD. Through the expansion of a pathogenic memory-activated T cell population, IL23 triggers an inflammatory cascade leading to intestinal inflammation. Using the TNBS-induced colitis animal model, we for the first time have identified miR-223 as a critical component of the IL23 inflammatory cascade in IBD. MiR-223 functions as a pro-inflammatory microRNA and is tightly controlled by IL23 in IBD. Pro-inflammatory miR-223 directly targets Claudin-8 (*CLDN8*), a critical family member in the maintenance of normal intestinal barrier property. Our study characterizes this novel IL23/miR-223/*CLDN8* pathway in the development of IBD (Fig. 7e).

TNBS-induced colitis is a well-established animal model to study mucosal inflammation for IBD pathogenesis and preclinical studies [26, 27]. In this study, we have used three parameters to give a comprehensive evaluation of the inflammation of colitis, including pathology score, MPO activity, and FITC permeability. The pathology score is a rough estimation of inflammation ranked by lab technicians. Although the therapeutic role of anti-IL23P19 has been well documented [28, 29], we found that the antibody treatment reduced the score in a very modest functional significance (Fig. 1d). In contrast, MPO is an enzyme found predominantly in the azurophilic granules of neutrophils as well as in monocytes and macrophages. Measurement of MPO activity is directly associated with neutrophil content, thus used as a quantitative index of inflammation in colitis [30]. Using this assay, we found a more statistical significance after the anti-IL23P19 treatment (Fig. 1f). Additionally, impaired epithelial barrier causes the increment in intestinal permeability. Quantitation of FITC permeability also gives a reliable estimation of colitis pathogenesis (Fig. 1e). Together, we demonstrate that the anti-IL23P19 treatment significantly alleviated colitis pathogenesis as previously reported [28, 29].

The intestinal epithelial barrier preserves the integrity of the intestine. The colonic mucosal barrier consists of a mucous layer and epithelial cells, which is the first physical barrier in intestinal innate immunity. A healthy barrier stops the invasion of pathogens and toxic substances, but remains tolerant to food antigens and normal microbiome [31–33]. Impaired epithelial barrier function causes increased intestinal permeability, triggering compensatory immune reactions, and a chronic inflammatory response. Recent murine and human studies have shown that defective barrier function is associated with IBD. Patients with IBD and their healthy relatives have increased intestinal permeability, suggesting that barrier dysfunction is an important feature contributing to inflammation in IBD [34, 35].

Claudins are a family of proteins that consists of more than 27 members. They were regarded as essential for maintenance of the intestinal barrier. Dysregulated claudins might lead to injury of the intestinal epithelial barrier. Some studies have found that dysregulated claudins were involved in CD patients. For example, Zeissig *et al.* reported that claudin-5 and *CLDN8* were downregulated in active CD patients, while claudin-2 was strongly upregulated. Other claudins such as claudin-1, 4, and 7 were unchanged [36]. Using integrated microarray analysis, Clark *et al.* found CLDN8 was highly downregulated in both CD and UC tissues [37]. Other studies have found that claudin-1, claudin-2, and claudin-4 expression were elevated in IBD patients [38, 39]. However, the role of claudins in the IL23 inflammatory cascade remains undefined. In this study, we show that *CLDN8* was downregulated in both IBD patients and in mice with TNBS-induced colitis under the control of miR-223. Decreasing *CLDN8* using siRNA reduced the TEER in Caco-2 cells. Ectopic expression of *CLDN8* enhanced the tight junction of epithelial cells. These data suggest that *CLDN8* might play an important role in the injury of intestinal epithelial barrier of IBD.

MiRNAs are thought to be involved in the pathogenesis of the inflammation in IBD. Using IL-10(−/−) mice as an animal model of Th1-mediated inflammatory bowel disease, Schaefer *et al.* showed that miR-223 was one of the miRNAs that was dysregulated in colonic tissues and PBLs of mice with mild intestinal pathology. The 3’ untranslated region of the Roquin ubiquitin ligase gene was a target for miR-223 [40]. Later, they also found that 26 miRNAs, including miR-223, were altered in colon biopsies of CD and UC patients [41]. Quite recently, Polytarchou *et al.* also showed that miR-223 was one of the 12 circulating microRNAs that differentiate patients with UC from control subjects [42]. In this study, we identified *CLDN8* as a downstream target of miR-223 in the IL23 pathway. Importantly, we demonstrate that knockdown of miR-223 restores *CLDN8* levels in mice with colitis and that it mitigates progression of colitis. Thus, miR-223 antagomir can ameliorate progression of colitis. MiR-223 may enhance the mucosal healing by repairing the injuries of intestinal epithelial barrier.

It should be emphasized that many other miRNAs, in addition to miR-223, may also be involved in the IL-23/Th17 pathway. For example, in this study we screened miRNAs that have been reported to be upregulated in IBD, including miR-223, miR-21, miR-155, miR-19a, miR-101, miR-594, and miR-16. Among them, miR-223 is the most upregulated miRNA. Additionally, a single miRNA may target tens to hundreds of distinct mRNAs, and an individual mRNA may be directly regulated by multiple miRNAs [43]. For example, Dorhoi *et al.* reported in tuberculosis that miR-223 directly targets the chemoattractants CXCL2, CCL3, and IL-6 in myeloid cells [44] and miR-223 downregulated the expression of STAT3 in sepsis [45]. Interestingly, by searching miR-223 targets (http://mirtarbase.mbc.nctu.edu.tw), it seems that some of the miR-223 targets are pro-inflammatory, indicating that miR-223 may be an anti-inflammatory microRNA. In our colitis model, however, we found that miR-223 is a pro-inflammatory miRNA. Similar pro-inflammatory role of miR-223 has been reported in other models [46–49]. Thus, future studies are needed to explore the miRNA/target RNA interactome network in the IL-23 pathway.

The approaches used to deliver drugs to animals with colitis include intraperitoneal [11, 50, 51], tail vein [52], or intracolonic administration [53]. By comparing intraperitoneal, caudal vein, and intracolonic delivery in our pilot studies, we were surprised to find that intraperitoneal delivery of anti-IL23P19 and antagomir223 was the most effective way to treat mice with TNBS-induced colitis. Theoretically, the intracolonic administration should be a more direct and colon-specific approach. However, intrarectal administration of TNBS during model establishment may damage the intestinal mucosa and thus influence drug absorption. Nonetheless, intracolonic administration of antagomir223 also alleviated TNBS-induced colitis (Additional file 6: Figure S4).

The IL23/Th17 pathway plays an important role in many autoimmune diseases including IBD [10]. Monoclonal antibodies targeting IL23p19 promote mucosal healing in experimental colitis [11]. In preclinical trials, Ustekinumab, a human monoclonal antibody against IL23, was effective in treating CD patients [54, 55]. A previous study demonstrates that IL–23 induces migration and invasion in thyroid cancer cells through the miR–25/SOCS4 signaling pathway [56]. In this study, we have made the novel observation that IL23 upregulates miR-223. Correspondingly, a monoclonal antibody that targets IL23 alleviates this process, leading to the downregulation of *CLDN8*. In experimental colitis, we found that the therapy that target the IL23/miR-223 pathway could decrease weight loss and improve histological appearance, histological score and MPO activity, and the integrity of the intestinal epithelial barrier.

## Conclusion

Our findings suggest a new mechanistic pathway in the IL23 cascade in IBD. MiR-223 functions as a pro-inflammatory molecule interacting with the IL23 pathway. By targeting *CLDN8*, miR-223 directly bridges the IL23 signal with intestinal barrier properties in IBD. Thus, disrupting this IL23/miR-223/*CLDN8* interaction may provide novel therapeutic strategies for the management of IBD.

## Methods

### Human IBD colon tissues

Colon biopsies were obtained from IBD patients and healthy volunteers during endoscopy at the First Affiliated Hospital, Sun Yat-sen University. Informed written consent was given by all the participants. The study protocol was approved by the Human Ethics Committee of the First Affiliated Hospital, Sun Yat-Sen University, and experimental methods comply with the Helsinki Declaration.

### Cell lines and culture conditions

SW480 (human colon adenocarcinoma), Caco-2 (human epithelial colorectal adenocarcinoma), and 293T (human embryonic kidney) cell lines were purchased from American Tissue Culture Collection (ATCC, VA, USA), and NCM460 (a human colon mucosal epithelial cell line) was purchased from Jennio Biotechnology (Guangzhou, China). All cells were cultured in 1640 medium supplemented with 10 % fetal bovine serum, penicillin, and streptomycin in a 5 % CO_2_ incubator.

### RNA preparation, Q-PCR for miRNA, and mRNA abundance

Total RNA was extracted from colon biopsies or cell lines using Trizol Reagent (Invitrogen, Carlsbad, CA, USA). The Transcriptor First Stand cDNA Synthesis Kit (Roche) and the Fast Start Universal SYBR Green Master (Roche) were used to confirm the miRNA and mRNA expression changes. The expression of each target miRNA and mRNA was calculated, respectively, relative to U6 or β-Actin. A comparative threshold cycle method was used to compare each condition with controls.

### Western blotting analysis

Total proteins were added to RIPA buffer (CST, USA) and boiled at 95 °C for 5 min. The proteins were then routinely processed for western blotting as described previously [57]. Briefly, the proteins were separated on 10 % SDS polyacrylamide gels and blotted onto nitrocellulose membranes which were incubated in TBST-milk, followed by primary antibodies (4 °C, overnight) for *CLDN8* IgG antibody (2 μg/mL, Gene Tex) (1:1,000 dilution) and GAPDH antibody (1:1,000, CST). Blots were then washed with TBST three times (10 min each) and subsequently incubated (1 h) with anti-rabbit IgG HRP-linked antibody (1:3,000, CST). Each western blot was repeated at least three times.

### Immunohistochemical staining (IHC)

The paraffin sections were deparaffinized in xylene and hydrated through a graded series of alcohol to tap water. Antigen retrieval was performed by microwave irradiation in citrate buffer for 20 min and cooled to room temperature. The sections were incubated with 3 % H_2_O_2_ in distilled water for 15 min to quench the endogenous peroxidase activity. After being rinsed three times with PBS, the sections were incubated with Rabbit polyclonal to *CLDN8* IgG antibody (2 μg/mL, Gene Tex) (1:500 dilution) overnight at 4 °C, and then washed in PBS; the sections were incubated with the secondary antibody (Dako) for 30 min at room temperature. Finally, the sections were counter stained with hematoxylin.

### Fluorescence *in situ* hybridization (FISH)

*In situ* hybridization for miR-223 and *CLDN8* was performed on paraffin sections of biopsies. The paraffin sections were deparaffinized in xylene and hydrated through a graded series of alcohol to tap water. Proteinase K digestion was used to treat fixed tissues at 37 °C for 5 min. After digestion, slides were immersed in RNase-free water for 3 min and then air dried. Hybridization was carried out overnight at 55 °C using hybridization buffer supplemented with denatured FITC-labeled Locked Nucleic Acid (LNA) probes (1:200, Exiqon) directed against miR-223. Scrambled LNA probes were used as a negative control. Simultaneous immunostainings were conducted using a rabbit polyclonal to *CLDN8* IgG antibody (2 μg/mL, Gene Tex) (1:500 dilution), followed by cy3-conjugated anti-rabbit secondary antibodies (1:400 dilution) for 30 min at room temperature. After washing, the sections were counterstained with 4’, 6-diamidino-2-phenylindole (DAPI) for 1 min. Images were captured with a Zeiss LSM710 confocal microscope (Zeiss, Oberkochen, Germany).

### CLDN8 3’-UTR construct and luciferase report assay

The 3’-UTR of *CLDN8* mRNA bearing miRNA binding sites (corresponding to 905–2147 nucleotides of RefSeq NM_199328.2) was cloned into the XhoI and NotI sites downstream of the Renilla luciferase reporter vector, pmiR-RB-REPORT (RiboBio, Guangzhou, China), according to the manufacturer’s instructions. The miR-RB-REPORT-mutated vector was generated corresponding to the predicted binding sites on the *CLDN8* 3’-UTR for miR-223. For the miRNA binding site, eight nucleotides in the 5’seeding region were substituted. Each pMIR construct (100 nM), along with the renilla luciferase control plasmid, phRL-CMV (Promega; 200 ng/well), was transfected into 293T cells in 24-well plates using Lipofectamine 2000 (Invitrogen) according to the manufacturer’s guidelines. Cells were harvested 48 h after transfection. Firefly and renilla luciferase activities were measured using the Dual Luciferase Reporter Assay System (Promega) according to the manufacturer’s instructions. Experiments were performed in triplicate.

### Knockdown of CLDN8 by siRNA and ectopic expression of CLDN8

We used predesigned siRNA for *CLDN8* as follows: 5’ GGGACAAUGAGAAGGUGAA dTdT 3’, 3’dTdT CCCUGUUACUCUUCCACUU 5’. Transient transfection of siRNA into Caco-2 and NCM460 cells was performed using Lipofectamine 2000 (Invitrogen) according to the manufacturer’s instructions. The ectopic expression of *CLDN8* with *CLDN8* plasmid (Vigenebio, CH855544) into the above cells was performed using a similar procedure.

### Transfection of miRNA mimics and inhibitors

The micrONTM miRNA mimics and inhibitors to miR-223 and the negative control #22 were obtained from RiboBio (Guangzhou, China). Caco-2, SW480, and NCM460 cells in 1640 medium containing 10 % fetal bovine serum were placed into 12-well plates at 50 % confluence at 37 °C in a 5 % CO_2_ incubator. After 24 h, the culture media was replaced with 1640 medium (without serum and antibiotics). MiRNA mimics, inhibitors, and the negative control were transiently transfected into the cells using Lipofetamine 2000 (Invitrogen, CA, USA) following the manufacturer’s instructions. The transfected cells were incubated at 37 °C in a 5 % CO_2_ incubator for 48 h for further study.

### Measurement of transepithelial electrical resistance (TEER)

TEER, a reliable indication of the tightness of junctions between colonic cells, was measured as described previously [58]. In brief, Caco-2 cells were seeded at a density of 2 × 10^5^cells/cm^2^ on the top of transwell polycarbonate filters (pore size, 3 μm; diameter, 24 mm; growth area, 4.5 cm^2^) from Costar (Millipore). Caco-2 monolayers were used 21 days after seeding. TEERs of the monolayers were measured using Millicell ERS-2 (Millipore, Germany).

### Cell treatment with Anti-IL23P19

Colonic epithelial cells were plated at density of 2 × 10^5^ cells per well in 6-well plates. Cells were treated for 0, 12, 24, 36, 48, 60, and 72 h with IL23 (20 ng/mL) with or without anti-IL23P19 neutralizating antibody (anti-IL23P19) (6 μg/mL) or its isotype control. We found that the expression of *CLDN8* decreased with time but became stabilized between 60 and 72 h (Additional file 7: Figure S5). Thus, a 72 h exposure was chosen for the study.

### TNBS-induced colitis model

Trinitrobenzene sulfonic acid (TNBS)-induced colitis are well-established models to study mucosal inflammation. The colitis model was established in the IBD center, First Affiliated Hospital, Sun Yat-sen University, following the procedure as previously reported [59]. Briefly, pathogen-free male BALB/c mice (6–8 weeks) were obtained from Slack Jingda Experimental Animal LTD of Hunan province (Hunan, China) and maintained under specific pathogenic-free conditions in the animal facilities of Sun Yat-Sen University. Animals were pre-sensitized with 1 % trinitrobenzene sulfonic acid (TNBS, Sigma, St Louis, MO, USA) at day 1, and received 2.5 % TNBS (mixing 1 volume of 5 % (w/v) TNBS with 1 volume of absolute ethanol) (2.5 mg/20 g, about 100 μL) intrarectally at day 8 [59].

In our pilot studies, we tested different doses of anti-IL23P19 and found that intraperitoneal injection of anti-IL23P19 at the dose of 0.5 mg/kg yielded the best therapeutic effect. This dose was thus used for the following study. Experimental animals were divided into three groups (10 mice per group). In Group 1, animals received TNBS dissolved in ethanol. Twenty-four hours after administration of TNBS, animals were treated with a neutralizing anti-mouse IL23 antibody (anti-IL23P19, Clone: G23-8; eBioscience) via intraperitoneal injection (0.5 mg/kg) for three consecutive days (TNBS + P19). In Group 2, after receiving TNBS, animals were treated with the isotype control (TNBS + ISO). In Group 3, animals received 100 μL of 50 % ethanol alone as the negative control (Control).

### Histological assessment of colitis

The colonic tissues of TNBS-colitis were removed, fixed in 10 % buffered formalin, embedded in paraffin, and tissue sections were stained with hematoxylin and eosin. The histology were scored blindly by lab technicians from Department of Pathology in the IBD-MDT center using previously described criteria [60]: 0, no signs of inflammation; 1, very low level; 2, low level of leukocyte infiltration; 3, high level of leukocyte infiltration, high vascular density, thickening of the colon wall; and 4, transmural infiltration, loss of globet cells, high vascular density, thickening of the colon wall.

### Myeloperoxidase (MPO) activity

The MPO activity assay, a biochemical assay for acute intestinal inflammation, was performed within 1 week of the collection of the colonic tissues according to the instructions of the MPO assay kit (Jiancheng BioEngineering, Nanjing, China). The absorbance was measured at 460 nm using a Life Science UV Spectrophotometer DU 530 (Beckman Coulter, USA). The MPO activity was expressed in units per gram of tissue, and 1 U corresponded to the activity required to degrade 1 mmol of hydrogen peroxide per minute at 25 °C.

### FITC-labeled dextran intestinal permeability assay

Intestinal permeability was examined on day 7 using the FITC-labeled dextran method, as previously described [61]. Briefly, mice were gavaged with 60 mg/100 g of FITC dextran (MW 4,000 at 80 mg/mL, Sigma) 4 h before sacrifice. Cardiac puncture was performed, blood was collected, and FITC concentrations were measured in plasma (Fluorimeter Pharos FX; BioRad, Hercules, CA, USA). A standard curve was obtained by diluting serial concentrations of FITC-Dextran in mouse serum. Serum analysis of FITC concentration was immediately performed in triplicate by using Spectra Max M5 (485/520 nm).

### Therapy of TNBS-induced colitis by miR-223 antagomir

Chemically modified miRNA antagomirs (Ambion, Austin, TX, USA) complementary to the mature miR-223 sequences were used to knock down miR-223 expression *in vivo*. The miR-223 antagomir was a 22-mer with the sequence 5’- UGUCAGUUUGUCAAAUACCCCA-3’, while the negative control sequence was a 21-mer with the sequence 5’-CAGTACTTTTGTGTAGTACAA-3’. All substances were dissolved at 3 mg/mL in RNase-free sterile PBS. In a pilot study, we compared the effect of different doses of antagomir-223 in our model (Additional file 5: Figure S3A). Based on these data, intraperitoneal injection of 7.5 mg/kg antagomir-223 was used for the following experiment.

The animals were divided into three groups. Two groups (10 mice for one group) were administrated with TNBS dissolved in ethanol, another group with ethanol alone. The two TNBS group were administrated with antagomir (7.5 mg/kg, intraperitoneal injection) and antagomir-control separately for three consecutive days 24 h after administration of TNBS. The mice were observed from day 1 to day 8. Body weights were monitored before the induction of colitis and daily thereafter. At the end of the experiment, the mice were sacrificed by cervical dislocation under anesthesia.

### Statistical analyses

Experimental results are expressed as mean ± SD. Statistical analyses for Q-PCR were performed with the unpaired, two-tailed Student’s t tests and one-way ANOVA for comparing all pairs of groups (SPSS 16.0). *P* <0.05 was considered significant.

## Additional files

Additional file 1: Table S1. Genes that showed greater than four-fold upregulation in IBD. (XLSX 28 kb) Additional file 2: Table S2. Genes that showed greater than four-fold downregulation in IBD. (XLSX 14 kb) Additional file 3: Figure S1. Identification of *CLDN8* as a novel target controlled by the IL23 pathway in IBD. **A** Heatmap of IBD-associated mRNAs. Lanes 4A-6A:CD tissues; lanes 4B-6B: normal colons. **B** Western blot of *CLDN8* in colonic inflamed mucosa of IBD patients. **C** Western blot of *CLDN8* in colonic inflamed mucosa of colitis mice. **D** Anti-IL23P19 restored *CLDN8* by western blot. (EPS 2313 kb) Additional file 4: Figure S2. Abundance of miR-223 in the mimic- and inhibitor-treated cells. **A** MiR-223 mimics upregulate miR-223 in colonic epithelial cells by qPCR. **B** MiR-223 inhibitors downregulate miR-223 in colonic epithelial cells by qPCR. **C** MiR-223-positive cells in colonic mucosa of IBD patients by FISH. **P* <0.05, ***P* <0.01, ****P* <0.001. (EPS 1921 kb) Additional file 5: Figure S3. Therapeutic treatment of miR-223 antagomir in mice with TNBS-induced colitis. **A** Dose-dependent study of miR-223 antagomir. **B** Representative images of the colon in treated mice with colitis. **C** Antagomir223 therapy reduces the histological score. **D** Effects on MPO activity measurement by antagomir223. **E** MiR-223 antagomirs downregulate miR-223. **P* <0.05, ***P* <0.01, ****P* <0.001. (EPS 4652 kb) Additional file 6: Figure S4. MiR-223 antagomirs therapy by direct intracolonic delivery. **A** Recovery of body weight in mice with colitis by miR-223 antagomirs. Control: colitis mice treated with ethanol; TNBS + Anti-miR: colitis mice treated with antagomir223; TNBS + Anti-CTL: colitis mice treated with an isotype control of antagomir223. **B** MiR-223 antagomirs therapy reduces the histological score. **C** Serum FITC-dextran was quantified as a measure of intestinal permeability. **D** Effects on MPO activity by miR-223 antagomirs. **E** MiR-223 antagomirs upregulate *CLDN8* as quantitated by qPCR. **F** MiR-223 antagomirs upregulate *CLDN8* as quantitated by western blot. **G** MiR-223 antagomirs downregulate miR-223. **P* <0.05, ***P* <0.01, ****P* <0.001. (EPS 5350 kb) Additional file 7: Figure S5. IL23 reduced the expression of CLDN8 in a time-dependent manner in NCM460 and Caco-2 cells. (EPS 758 kb)

## Abbreviations

CD: Crohn’s disease
FISH: fluorescence *in situ* hybridization
IBD: inflammatory bowel disease
IHC: immunohistochemistry
JAM: junctional adhesion molecule
MPO: Myeloperoxidase
TEER: transepithelial electrical resistance
TJs: tight junctions
TNBS: 2,4,6-trinitrobenzene sulfonic acid
UC: ulcerative colitis
